# Determinants of completing recommended antenatal care utilization in sub-Saharan from 2006 to 2018: evidence from 36 countries using Demographic and Health Surveys

**DOI:** 10.1186/s12884-021-03669-w

**Published:** 2021-03-06

**Authors:** Zemenu Tadesse Tessema, Achamyeleh Birhanu Teshale, Getayeneh Antehunegn Tesema, Koku Sisay Tamirat

**Affiliations:** Department of Epidemiology and Biostatistics, Institute of Public Health, College of Medicine and Health Sciences, University of Gondar, Gondar, Ethiopia

**Keywords:** Recommended ANC utilization, SSA, Determinants, The pooled prevalence

## Abstract

**Background:**

Every day in 2017, approximately 810 women died from preventable causes related to pregnancy and childbirth, with 99% of these maternal deaths occurring in low and lower-middle-income countries. Sub-Saharan Africa (SSA) alone accounts for roughly 66%. If pregnant women gained recommended ANC (Antenatal Care), these maternal deaths could be prevented. Still, many women lack recommended ANC in sub-Saharan Africa. This study aimed at determining the pooled prevalence and determinants of recommended ANC utilization in SSA.

**Methods:**

We used the most recent standard demographic and health survey data from the period of 2006 to 2018 for 36 SSA countries. A total of 260,572 women who had at least one live birth 5 years preceding the survey were included in this study. A meta-analysis of DHS data of the Sub-Saharan countries was conducted to generate pooled prevalence, and a forest plot was used to present it. A multilevel multivariable logistic regression model was fitted to identify determinants of recommended ANC utilization. The AOR (Adjusted Odds Ratio) with their 95% CI and *p*-value ≤0.05 was used to declare the recommended ANC utilization determinates.

**Results:**

The pooled prevalence of recommended antenatal care utilization in sub-Saharan Africa countries were 58.53% [95% CI: 58.35, 58.71], with the highest recommended ANC utilization in the Southern Region of Africa (78.86%) and the low recommended ANC utilization in Eastern Regions of Africa (53.39%). In the multilevel multivariable logistic regression model region, residence, literacy level, maternal education, husband education, maternal occupation, women health care decision autonomy, wealth index, media exposure, accessing health care, wanted pregnancy, contraceptive use, and birth order were determinants of recommended ANC utilization in Sub-Saharan Africa.

**Conclusion:**

The coverage of recommended ANC service utilization was with high disparities among the region. Being a rural residence, illiterate, low education level, had no occupation, low women autonomy, low socioeconomic status, not exposed to media, a big problem to access health care, unplanned pregnancy, not use of contraceptive were determinants of women that had no recommended ANC utilization in SSA. This study evidenced the existence of a wide gap between SSA regions and countries. Special attention is required to improve health accessibility, utilization, and quality of maternal health services.

## Background

Every day in 2017, approximately 810 women died from preventable causes related to pregnancy and childbirth [1]. Countries in Sub-Saharan Africa (SSA) and Southern Asia covers approximately 86% (254000) of the estimated global maternal deaths in 2017, with sub-Saharan Africa alone accounting for roughly 66% (196000), and Southern Asia accounted for nearly 20% (58000) [2]. Although by 2015, maternal mortality had decreased by over 40% from the 1990 levels, maternal death rate continued to remain unacceptably high in SSA [1]. The Sustainable Development Goals (SDGs) create a transformative new strategy for maternal health for ending preventable maternal mortality; aim 3.1 of SDG 3 is to reduce the global MMR to less than 70 per 100,000 live births by 2030 [3]. Hemorrhage is the leading cause of maternal mortality, accounting for over one quarter (27%) of deaths. A comparable proportion of maternal deaths is caused indirectly by pre-existing medical problems exacerbated by the birth. Hypertensive pregnancy disorders, particularly eclampsia, sepsis, embolism, and complications of unsafe abortion, also claim a significant number of lives [4]. Besides, not accessing quality antenatal care (ANC) leads substantially to these preventable maternal deaths [5, 6].

Between 2000 and 2017, the global maternal mortality ratio decreased by 38%, and the estimated annual rate of decline in MMR between 2000 and 2017 was 2.9 [7]. The high rate of maternal deaths in certain parts of the world represents gaps in access to quality health care and highlights the difference between rich and poor. The MMR in low-income countries in 2017 is 462 per 100,000 live births in high-income countries [8].

There is a large variance between developed (98%) and low-income countries (68%) countries in its coverage. Although ANC services’ coverage is growing in many African countries, coverage alone does not provide adequate details on the service [9]. There is a substantial-quality deficit in ANC facilities in sub-Saharan Africa. Although availability with at least one ANC appointment is comparatively high at 71%, many women attending ANC do not access the full spectrum of evidence-based components during pregnancy. This consistency discrepancy highlights crucial missed opportunities inside health systems [10, 11].

Different scholars identified factors associated with recommended antenatal care utilization. A systematic review conducted in Sub-Saharan Africa identified residence, age, parity, education level, employment status, marital status, and religious factors associated with recommended ANC utilization [12–16]. Another study conducted in Africa identified age, marital status, occupation, residence, distance to the health facility, and parity were factors associated with recommended ANC utilization [16]; studies conducted in sub-Sahara Africa identified age, residence parity, and geographic location as factors associated with recommended ANC visit [9–11, 17, 18].

Even though several multilevel studies have been conducted to determine determinants associated with recommended antenatal care utilization in the SSA region, no multicounty study incorporated all SSA countries that had DHS data. This study includes all SSA countries that had DHS data that would give a generalizable and reliable estimate. This study would help policy and decision-makers effectively plan resources in regions where low recommended ANC utilization.

## Method

### Data source

Thirty-six sub-Saharan Africa countries’ most recent Demographic and Health Surveys (DHS) data were used for this study (Table 1). The countries were given a unique identification number and appended together to have a single dataset that represents the sub-Saharan Africa countries. The DHS dataset is representative of each nation in the sub-Saharan Africa countries. The detail of the DHS dataset was found from our previously published work [19].

**Table 1:** Pooled Demographic and Health Surveys (DHS) data from 36 sub-Saharan countries, 2006–2018

Country	DHS year	Sample size (260,572)
**Southern Region of Africa**		**14,294**
Lesotho	2014	8409
Namibia	2013	11,002
Swaziland	2006/07	5851
South Africa	2016	2935
**Central Region of Africa**		**48,651**
Angola	2015/16	8909
DR Congo	2013/14	11,002
Congo	2011/12	5851
Cameroon	2011	7564
Gabon	2012	3635
Sao Tome & Principe	2008/09	1282
Chad	2014/15	10,906
**Eastern Region of Arica**		**99,924**
Burundi	2010	8934
Ethiopia	2016	7574
Kenya	2014	14,396
Comoros	2012	1758
Madagascar	2008/09	8571
Malawi	2015/16	13,463
Mozambique	2011	7787
Rwanda	2014/15	6060
Tanzania	2015/16	7043
Uganda	2011	10,096
Zambia	2018	7262
Zimbabwe	2013/14	4973
**Western Region of Africa**		**260,572**
Burkina-Faso	2010	10,478
Benin	2017	8798
Cote d’Ivoire	2011	5199
Ghana	2014	4120
Gambia	2013	5298
Guinea	2018	5339
Liberia	2013	4606
Mali	2018	6463
Nigeria	2018	21,552
Niger	2012	7962
Sierra Leone	2010/11	7532
Senegal	2010/11	7503
Togo	2013/14	4839

The DHS data had different datasets. For this study, Individual records (IR dataset) were used. The dataset includes marriage and sexual activity, fertility, fertility preference, family planning, anthropometry and anemia in women, malaria prevention for women, HIV/AIDS, women’s empowerment, adult and maternal mortality, and domestic violence. The detail of the dataset was published elsewhere [20].

The two-stage stratified sampling technique was used to select the study participants in the DHS dataset. We appended 36 subiSaharan Africa countries after unique IDs were given for each country. Pooled analysis was done after sampling weight. A total of 260,572 reproductive-age women who gave at least one birth in the 5 years preceding each country survey was included in this study.

### Variables

#### Outcome variable

The outcome variable for this study was whether a woman had four and above antenatal care visits or not. The variable is generated using WHO-recommended antenatal Care service. We coded “1” if women had four and above antenatal care visit service and”0″ otherwise [9].

#### Explanatory variables

Based on known facts and literature [17, 21–23], the explanatory variables included in this study were region, residence, age group, maternal education, husband education, maternal occupational status, women autonomy on health care, wealth index, media exposure, accessing health care, wanted pregnancy, contraceptive utilization, and birth order.

#### Theoretical framework

**Fig. 1:**
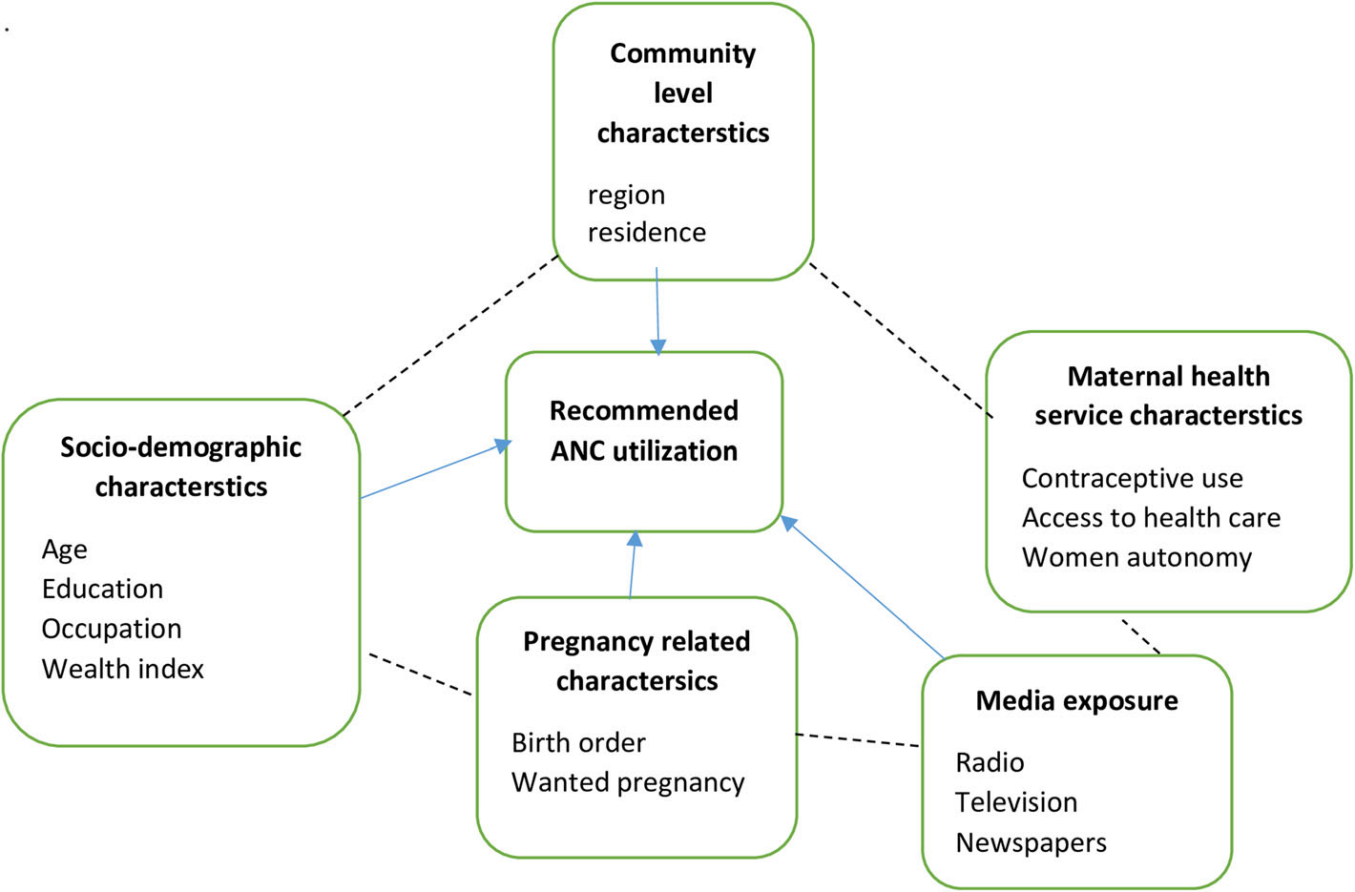
Theoretical review of the relationship between recommended ANC utilization and variables in SSA from 2006 to 2018

The theoretical literature review help establish what theories already exist, the relationships between them, to what degree the existing theories have been investigated, and to develop new hypotheses to be tested. The following diagram was created to clearly define the relationship between recommended ANC utilization and variables using solid and broken lines. The solid line indicates a direct relationship, and the broken line indicates an indirect relationship. The figure presented that factors such as community-level characteristics, socio-demographic characteristics, pregnancy-related characteristics, media exposure, and maternal health service characteristics could affect recommended ANC utilization. More ever, the figure illustrated the theoretical relationship between recommended ANC utilization across sub-Saharan Africa countries (Fig. 1).

#### Data management and analysis

The data were weighted using sampling weight, primary sampling unit, and strata before any statistical analysis to restore the representativeness of the survey and tell the STATA to consider the sampling design when calculating standard errors to get reliable statistical estimates. Descriptive and summary statistics were conducted using STATA version 14 software. The pooled prevalence of antenatal care utilization with a 95% Confidence Interval (CI) was reported for sub- Saharan Africa Countries from 2006 to 2018. The detail of the data management was found from our previously published work [19].

#### Statistical modeling

The DHS data had a hierarchical structure, which violates the independent assumptions. Women are nested within clusters, and women within the same cluster are more similar than the rest of the cluster. This nature of the DHS data needs to take into account the between cluster variability using appropriate statistical modeling.

Four models were fitted null model (models without the explanatory variables), a model I (models include community-level variables, model II (models include individual-level variable)) and Model III (models include both individual and community level variables) were fitted to select the best fit model for the data using Log-Likelihood Ratio (LLR) and Deviance [24, 25]. Model III, which includes both individual and community level variable, was selected because of its highest LLR and Smallest deviance (Table 3**).**

#### Fixed and random effect estimates

The fixed effect analysis was done using included variables in the model, both individual and community-level variables. The random effect analysis was done by considering variations between clusters (EAs) assessed by computing the Intra-class correlation coefficient (ICC), a proportional change in variance (PCV), and median odds ratio (MOR) [25–27]. The ICC is the proportion of variance explained by the grouping structure in the population. It was computed as ICC= $\frac{{\sigma_{\mu}}^2}{{\sigma_{\mu}}^2+{\pi}^2/3\operatorname{}}$; Where: the standard logit distribution has a variance of ${\pi}^2\kern-0.17em /\kern-0.17em 3\operatorname{}$, *σ*_*μ*_^2^ indicates the cluster variance. Whereas PCV measures the total variation attributed by individual level and community level factors in the multilevel model as compared to the null model. It was computed as: $\frac{\mathrm{varianceofnullmodel}-\mathrm{varianceoffullmodel}}{\mathrm{varianceofnullmodel}}$. MOR is defined as the odds ratio’s median value between the cluster at high risk and cluster at lower risk of recommended ANC utilization when randomly picking out two clusters (EAs). It was computed as: MOR = exp. ($\sqrt{2\ast {\sigma_{\mu}}^2\ast 0.6745}$) ~ MOR = exp. (0.95 ∗ σ_μ_).

#### Ethics consideration

Permission to get access to the data was obtained from the measure DHS program online request from http://www.dhsprogram.com.website, and the data used were publicly available with no personal identifier.

## Result

### Descriptive characteristics of the study participants

A total of **260,572** women who gave birth at least once 5 years preceding the survey were included. Of these, the largest study participants, 99,701(38.26%), were from Western Africa Region, and the smallest study participants, 14,294(5.49%), were from Southern Regions of Africa. The majority of study participants, 173,833(66.71%), were rural residents. The median age of women included in his study was 28.8 (IQR = 7.2) years, of which 119,146(45.72%) of them under the age category 25–34. Thirty-seven percent of women and 36 % of men had no formal education. More than one-third of women, 110,745(42.50%), were under poor wealth status **(**Table 2**).**

**Table 2:** Distribution of recommended ANC utilization in sub-Saharan Africa region from 2006 to 2018

Variable	Recommended ANC Utilization		Total sample size (%)	X-square value	p-value
	Yes	No			
**Africa Region**					
Southern	11,252	3042	14,294 (5.49)	60.25	< 0.001*
Central	27,256	21,395	48,651 (18.67)		
Eastern	52,006	45,917	97,924 (37.58)		
Western	55,078	44,622	99,701 (38.26)		
**Residence**					
Rural	84,114	89,718	173,833 (66.71)	160.68	< 0.001*
Urban	61,478	25,259	86,738 (33.29)		
**Age group**					
15–24	48,845	35,484	79,329 (30.44)	103.50	< 0.001*
25–34	67,949	51,196	119,146 (45.72)		
35–46	33,798	28,297	62,095 (23.83)		
**Marital status**					
Single	11,929	6643	18,573 (7.13)	576.50	< 0.001
Married	133,664	108,334	241,998 (92.87)		
**Literacy**					
Cannot read and write	58,984	71,010	129,905 (49.85)	60.05	< 0.001
Can read and write	86,699	43,967	130,666 (50.51)		
**Maternal education**					
No education	40,215	56,630	96,845 (37.17)	70.97	< 0.001*
Primary education	49,432	39,760	89,193 (34.23)		
Secondary and above	55,945	18,586	74,532 (28.60)		
**Husband education**					
No education	35,000	48,442	83,442 (36.89)	196.83	< 0.001*
Primary education	33,200	30,177	63,377 (28.02)		
Secondary and above	56,470	22,908	79,378 (35.09)		
**Maternal Occupation**					
Had occupation	108,220	84,024	68,327 (26.22)	126.12	< 0.001*
Had no occupation	37,373	84,024	192,244 (73.78)		
**Women’s health care decision making autonomy**					
Women alone	21,008	13,807	34,815 (13.36)	32.12	< 0.001
Women and her husband	48,307	32,120	80,427 (30.87)		
Husbands alone	76,287	69,050	145,329 (55.77)		
**Wealth Index**					
Poor	51,171	59,574	110,745 (42.50)	144.61	< 0.001*
Middle	28,833	23,455	52,288 (20.07)		
Rich	65,588	31,948	97,537 (37.43)		
**Media Exposed**					
Yes	107,961	65,824	173,786 (66.69)	158.67	< 0.001*
No	37,632	49,153	86,785 (33.31)		
**Accessing health care**					
Big problem	79,825	73,713	153,538 (58.92)	458.11	< 0.001*
Not big problem	65,768	41,264	107,033 (41.08)		
**Wanted pregnancy**					
Yes	132,528	103,876	236,404 (93.47)	37.33	< 0.001
No	8708	7799	16,508 (6.53)		
**Contraceptive use**					
Yes	53,142	27,567	179,862 (69.03)	102.43	< 0.001*
No	92,451	87,410	80,709 (30.97)		
**Birth Order**					
1	34,600	20,327	54,928 (21.08)	537.22	< 0.001*
2–4	71,536	52,160	123,696 (47.47)		
5+	39,457	42,489	81,946 (31.45)		

* = significant association between recommended ANC and independent variables

### Pooled prevalence of recommended antenatal care utilization

**Fig. 2:**
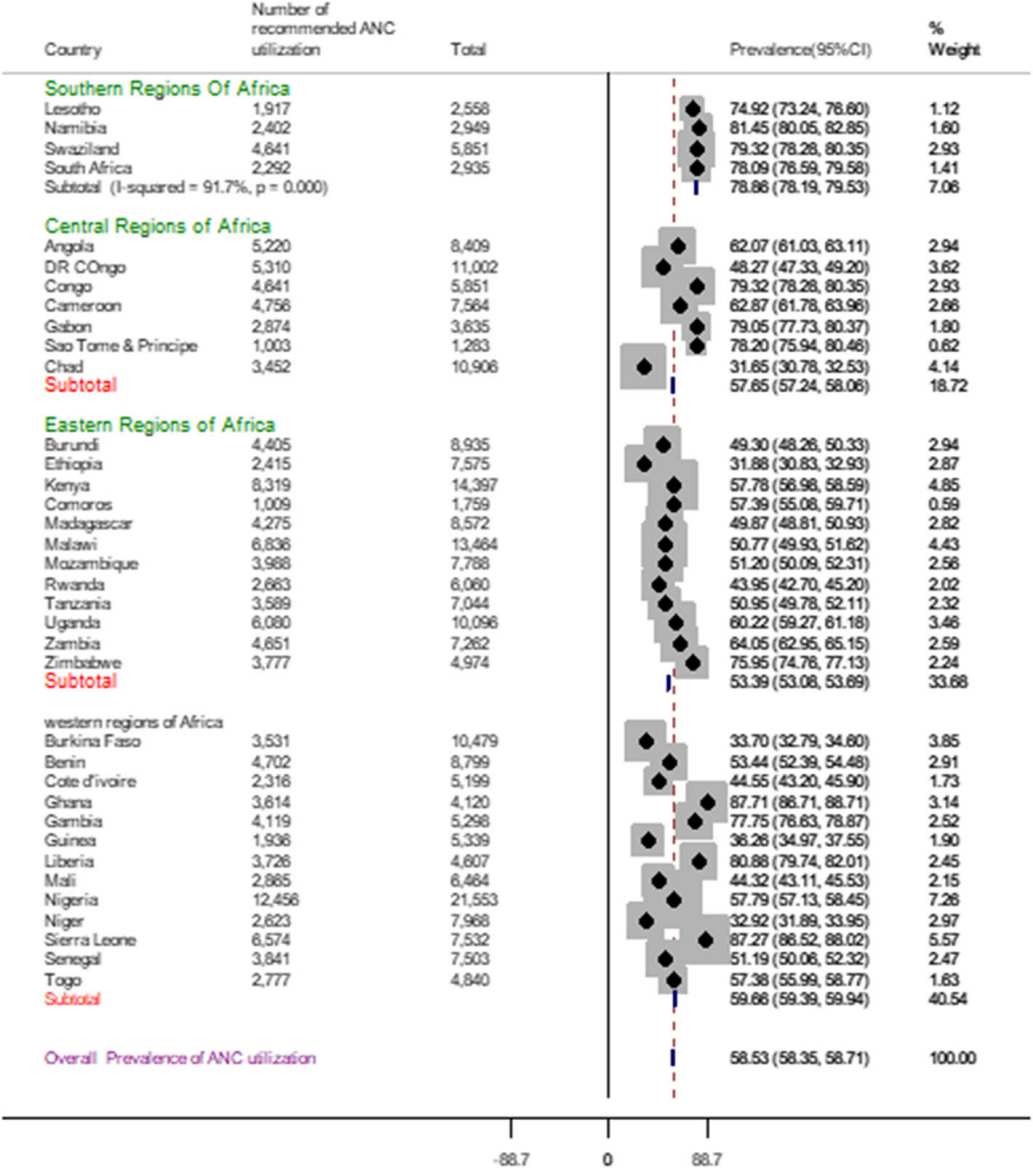
Forest plot of recommended antenatal care utilization in Sub-Saharan Africa from 2006 to 2018

The pooled prevalence of recommended antenatal care utilization in sub-Saharan Africa countries was 58.53% [95% CI: 58.35, 58.71], with the highest recommended ANC utilization in the Southern Region of Africa (78.86%) and the low recommended ANC utilization in Eastern Regions of Africa (53.39%). The sub-group analysis result evidenced that in Southern regions of Africa highest recommended ANC utilization, 79.32% were recorded in Swaziland, and the lowest recommended ANC utilization, 74.92% were recorded in Lesotho. In the Central Regions of Africa highest recommended ANC utilization, 79.32% were recorded in Congo, and the lowest recommended ANC utilization, 31.65%, was from Chad. In Eastern regions of Africa highest recommended ANC utilization, 75.95% were recorded in Zimbabwe, and the lowest recommended ANC utilization, 31.88%, was from Ethiopia. In the Western Regions of Africa, the highest recommended ANC utilization, 87.71% were from Ghana, and the lowest recommended ANC utilization, 32.92% were from Niger (Fig. 2).

### Determinants of recommended antenatal care utilization

The model fitted for this study was multilevel multivariable logistic regression. There are two parts of estimates in this model: the random-effects estimates and fixed estimate. The fixed and random effect estimates were observed by fitting four models (Null model, Model I, Model II, Model III). The empty model shows that there was significant variation in the likelihood of recommended ANC utilization within sub-Saharan Africa Countries (ϭ^2^ = 0.131, *p* < 0.001). The ICC in the empty model implied that 38% of the recommended ANC utilization variation contributed to the difference between Countries. The cluster-level variance was expressed as ICC and MOR. Moreover, the MOR was 1.21 (95%CI:1.19,1.23), which implies that the odds of recommended ANC utilization was 1.21 times more likely when women go from low recommended ANC utilization area to high recommended ANC utilization areas. In model III (full model adjusted for individual and community level factors), cluster level variance (ϭ^2^ = 0.041, p < 0.001) remained significant. A total of 68.70% variability in recommended ANC utilization that can be contributed to the country level factors was observed. The proportional change in variance (PCV) in this model was 68.7%, which indicated 68.70% of cluster variance observed in the empty model was explained by both community and individual level variable (Table 3).

**Table 3:** Multilevel multivariable logistic regression model analysis result of recommended antenatal care visit in Sub-Saharan Africa from 2006 to 2018

Variable	Null Model AOR(95%CI)	Model I AOR(95%CI)	Model II AOR(95%CI)	Model III AOR(95%CI)
**Africa Region**				
Southern		1		1
Central		0.33 (0.31,0.34)		0.50 (0.48,0.53)*
Eastern		0.37 (0.35,0.39)		0.46 (0.44,0.48)*
Western		0.36 (0.34,0.37)		0.72 (0.68,0.75)*
**Residence**				
Rural		1		1
Urban		2.21 (2.17,2.25)		1.35 (1.31,1.38)*
**Age group**				
15–24			1	1
25–34			1.27 (1.24,1.30)	1.24 (1.21,1.27)*
35–46			1.49 (1.44,1.53)	1.43 (1.38,1.48)*
**Literacy rate**				
Cannot read and write			1	1
Can read and write			0.93 (0.91,0.96)	1.008 (0.98,1.03)
**Maternal education**				
No education			1	1
Primary education			1.37 (1.33,1.41)	1.45 (1.41,1.49)*
Secondary and above			2.14 (2.06,2.23)	2.02 (1.94,2.10)*
**Husband education**				
No education			1	1
Primary education			1.15 (1.12,1.18)	1.29 (1.26,1.32)*
Secondary and above			1.67 (1.63,1.72)	1.70 (1.65,1.74)*
**Maternal Occupation**				
Had no occupation			1	1
Had occupation			1.11 (1.09,1.14)	1.12 (1.09,1.14)*
**Women’s health care decision making autonomy**				
Women alone			1.17 (1.14,1.20)	1.26 (1.23,1.30)*
Women and her husband			1.23 (1.21,1.26)	1.32 (1.29,1.35)*
Husbands alone			1	1
**Wealth Index**				
Poor			1	1
Middle			1.11 (1.09,1.14)	1.09 (1.06,1.11)*
Rich			1.26 (1.23,1.29)	1.13 (1.10,1.16)*
**Media Exposed**				
No			1	1
Yes			1.44 (1.41,1.47)	1.33 (1.30,1.36)*
**Accessing health care**				
Big problem			1	1
Not big problem			1.22 (1.20,1.25)	1.19 (1.17,1.25)*
**Wanted pregnancy**				
No			1	1
Yes			1.19 (1.15,1.24)	1.18 (1.14,1.23)*
**Contraceptive use**				
No			1	1
Yes			1.31 (1.28,1.34)	1.34 (1.32,1.37)*
**Birth Order**				
1			1	1
2–4			0.79 (0.77,0.82)	0.78 (0.77,0.82)*
5+			0.64 (0.61,0.66)	0.66 (0.63,0.68)*
Community variance (SE)	0.131 (0.112,0.153)	0.099 (0.084,0.117)	0.05 (0.042,0.060)	0.041 (0.034,0.049)
ICC%	38 (33,44)	29 (24,34)	15 (12,17)	12 (10,14)
PCV%	1	24.42	61.83	68.70
MOR	1.41 (1.37,1.45)	1.34 (1.31,1.38)	1.23 (1.21,1.26)	1.21 (1.19,1.23)
LL	− 180,239	− 174,792	− 143,943	−142,542
Deviance	360,478	349,584	287,886	285,084
AIC	360,483	349,596	287,927	285,133
BIC	360,504	549,658	288,134	285,381

* = significant at alpha 5%

In the multilevel multivariable logistic regression model; Sub-Sahara Africa region, residence, literacy level, maternal education, husband education, maternal occupation, women health care decision autonomy, wealth index, media exposure, contraceptive use, birth order, and wanted pregnancy were statistically associated with recommended ANC utilization in Sub-Saharan Africa.

Women living in Central, Eastern and Western Regions of Africa have decrease odds of recommended ANC utilization by 50, 54, and 28% as compared to women living in South Regions of Africa (AOR = 0.50, 95% CI: 0.48, 0.53), (AOR = 0.46, 95% CI: 0.44, 0.48) and (AOR = 0.46, 95% CI: 0.44, 0.48) respectively. The odds of ANC utilization among urban women increased by 35% compared to rural women (AOR = 1.35, 95% CI: 1.31, 1.38).

The odds of recommended ANC utilization among women age group 25–34 and 35–49 increase by 24% (AOR = 1.24, 95% CI: 2.11, 2.28) and 43% (AOR = 1.45, 95% CI: 1.38, 1.48) as compared to women age group 15–24 respectively. The odds of recommended ANC utilization among women who had primary and secondary and above educational level were 1.45 (AOR = 1.45, 95% CI: 1.41, 1.49) and 2.02 (AOR = 2.02, 95% CI: 1.94, 2.10) times higher as compared to women who had no formal education. The odds of recommended ANC utilization among women whose husband had primary and secondary and above educational level were 1.29 (AOR = 1.29, 95% CI: 1.26, 1.32) and 1.70 (AOR = 1.70, 95% CI: 1.65, 1.74) times higher as compared to women whose husband had no formal education. Women who had occupation were 1.12 (AOR = 1.09, 95% CI: 1.09, 1.14) times more likely to utilize recommended ANC than women who had no occupation. The odds of recommended ANC utilization among women who can decide health care service by themselves and with their husband increase by 26% (AOR = 1.26, 95% CI: 1.23, 2.30) and 32% (AOR = 1.32, 95% CI: 1.29, 1.35) as compared to women whose health care utilization decided by their husband alone. Women whose wealth status middle and rich were 1.09 (AOR = 1.09, 95% CI: 1.06, 1.11) and 1.13 (AOR = 1.38, 95% CI: 1.32, 1.43) times more likely to utilize recommended ANC than poor women. The odds of recommended ANC utilization among media exposed women were 1.33 times higher than women who were not exposed to media (AOR = 1.33, 95% CI: 1.30, 1.36). Women who reported accessing health care not a big problem were 1.19(AOR = 1.19, 95% CI: 1.17, 1.25) more likely to utilize recommended ANC than women who reported accessing health care big problem. Women who had wanted pregnancy were 1.18(AOR = 1.18, 95% CI: 1.14, 1.23) times more likely to utilize recommended ANC than those who did not want pregnancy. Women who use contraceptives were 1.34(AOR = 1.34, 95% CI: 1.32, 1.37) times more likely to utilize recommended ANC than Women who did not use a contraceptive. The odds of ANC utilization among women whose birth order 2–4 and 5+ were decreased by 22% (AOR = 0.78, 95% CI: 0.77, 0.82) and 34%(AOR = 0.66, 95% CI: 0.63, 0.68) as compared to women who had first birth order. (Table 3).

## Discussion

This study revealed that the recommended ANC utilization in the SSA region is low. The pooled prevalence recommended ANC visit was presented using the forest plot. Determinants of ANC utilization in SSA were identified by using a multilevel logistic regression model. As a result, urban residence, better maternal education, better husband education, had maternal occupation, women health care decision autonomy, better wealth status, had media exposure, accessing health care not a big problem, wanted pregnancy, contraceptive use was positively associated with recommended ANC utilization. In contrast, birth order was negatively associated with recommended ANC utilization.

The pooled prevalence of recommended antenatal care utilization in sub-Saharan Africa countries was 58.53% [95% CI: 58.35, 58.71], with the highest recommended ANC utilization in the Southern Region of Africa (78.86%) and the lowest recommended ANC utilization in Eastern Regions of Africa (53.39%). This finding was lower than a systematic review and meta-analysis conducted in Ethiopia at 63.77% [5], Ghana 86% [14], Liberia 76.13% [28], Angola 82.5% [22]. The possible justification for this discrepancy might be the systematic review and meta-analysis studies; there is a sample size issue and quality of articles that include the meta-analysis. Other studies are single-country study and not representative of other the region. The African Region has large intraregional disparities in terms of coverage of basic maternal health interventions like antenatal care. The lowest recommended ANC utilization was recorded in the East Africa Region, which faced decades of political instability, conflicts, poor quality of healthcare governance, inadequate health financing and human health resources, low standard health service delivery, and poor socioeconomic status [29, 30]. The best recommended ANC utilization was recorded in the Southern region of Africa. The Southern Africa region reported almost universal coverage in 2010; in West Africa, about one-third of pregnant women did not receive antenatal care visits [31].

The odds of using recommended ANC utilization increases among urban women. This finding is similar concepts to studies conducted elsewhere [15, 18, 21, 23, 32]. Inequalities could explain this difference in service accessibility and women’s awareness of ANC services in the rural setup compared with urban counterparts [33].

The odds of recommended ANC utilization increases among women and her partner who had better education levels. This result has similar findings with other previous studies [5, 13, 14, 16, 33]. This is because education has a positive impact on health service utilization and increment of knowledge about specific issues. Empowering women through education, household wealth, and decision-making increases maternal healthcare service utilization [34].

Our findings suggest that women’s occupation influences the completeness of antenatal care visits in the region. This result has similar findings with other previous studies [35, 36]. These findings may be related to both income and societal influences that come with employment outside of the home.

This study revealed that the likelihood of recommended ANC utilization increases among women who had autonomy on maternal health service utilization by themselves as compared to women who had partial autonomy (decided with her husband and husband alone). This result has similar findings with other previous studies [37–39]. The is due to women’s autonomy with respect to health care utilization enables women decision-making in obtaining health care for themselves [40].

This study showed that women with better economic status (wealth index) were increased on the number of antenatal care visit compared to poor wealth index. The possible reason might be mothers with better economic ststus can pay for health service costs such as transportation, medications, and any associated costs and can easily get information about the benefit of completing the ANC visits. This finding is supported by studies conducted elewhere [18, 41].

This study evidenced that the odds of using recommended ANC utilization among women who were exposed to mass media were higher than women who had no exposure to mass media. This could be because mass media can reach many people at a time and increase knowledge by changing family behavior on maternal health service and its advantage. This finding was also supported by studies conducted elsewhere [15, 42].

The finding also revealed that access to health care had a significant role in antenatal care visits. Completing four or more ANC visits among women who face accessing health care were less likely than women who did not face health care access challenges. This is because assessing health care services is important for promoting and maintaining their health, reducing unnecessary disability, and premature death. In addition, accessibility is related to transport issues, financial burden, and long distance to the health facility [15, 43, 44].

This study evidenced that women who had planned pregnancy were more likely to had recommended ANC visit compated to its counterpart. The implication might be unplanned pregnancy might unwilling to seek ANC visit. Besides, the absence of a pregnancy ‘mindset,’ which is common in unexpected or unplanned pregnancy, could have exerted a negative influence on mothers’s use of ANC services, and mother who plans to have a child might want to have a healthy pregnancy and thus might give great attention for their antenatal care service. This finding is supported by studies conducted elsewhere [5, 45].

Completing four or more ANC visits among mothers who use modern family planning was more likely to complete than the others. This is because those mothers who were using family planning might have a probability of more awareness and knowledge about the health providers’ maternal health services. During counseling, family planning could be increased in women’s knowledge about available family planning services and the medical facilities that provide such services. This finding is supported by studies conducted elsewhere [43, 46]. Birth order was a significant determinant of recommended ANC service utilization in sub-Saharan Africa. This is in line with previous studies [17]. Birth limiting can help to enhance maternal health service utilization as well as to improve maternal health.

### Strength and limitation of the study

Findings from the study are supported by large datasets covering 36 countries in SSA. The data were gathered following a common internationally acceptable methodological procedure. Due to the representative nature of the survey, the findings are representative of included countries and generalizable to women in SSA. The DHS survey year variation may affect this result. The data was collected based on self-reports from mothers within 5 years preceding the survey, which could be a potential source of recall and misclassification bias.

## Conclusion

The utlization of recommended ANC service low with high varation among the region. Being a rural residence, illiterate, low education level, had no occupation, low women autonomy, low socioeconomic status, not exposed to media, a big problem to access health care, unplanned pregnancy, not use of contraceptive were determinants of women that had no recommended ANC utilization in SSA. This study evidenced the existence of a wide gap between SSA regions and countries. Special attention is required to improve health accessibility, utilization, and quality of maternal health services.

## Data Availability

Data is available online and you can access it from www.measuredhs.com.

## Abbreviations

ANC: Antenatal Care
AOR: Adjusted Odds Ratio
CI: Confidence Interval
DHS: Demographic Health Survey
ICC: Intra-class Correlation Coefficient
LLR: log-likelihood Ratio
LR: Likelihood Ratio
MOR: Median Odds Ratio
SSA: Sub-Saharan Africa
WHO: World Health Organization
